# Comparative analysis on the expression of L1 loci using various RNA-Seq preparations

**DOI:** 10.1186/s13100-019-0194-z

**Published:** 2020-01-06

**Authors:** Tiffany Kaul, Maria E. Morales, Alton O. Sartor, Victoria P. Belancio, Prescott Deininger

**Affiliations:** 1 Tulane Cancer Center, Tulane Health Sciences Center, 1700 Tulane Ave, New Orleans, LA 70112 USA; 2 Section of Hematology and Oncology, Department of Medicine, Tulane School of Medicine, 1430 Tulane Ave, New Orleans, LA 70112 USA; 3 Department of Structural and Cellular Biology, Tulane School of Medicine, 1430 Tulane Ave, New Orleans, 70112 USA; 4 Department of Epidemiology, Tulane School of Public Health and Tropical Medicine, New Orleans, LA 70112 USA

## Abstract

**Background:**

Retrotransposons are one of the oldest evolutionary forces shaping mammalian genomes, with the ability to mobilize from one genomic location to another. This mobilization is also a significant factor in human disease. The only autonomous human retroelement, L1, has propagated to make up 17% of the human genome, accumulating over 500,000 copies. The majority of these loci are truncated or defective with only a few reported to remain capable of retrotransposition. We have previously published a strand-specific RNA-Seq bioinformatics approach to stringently identify at the locus-specific level the few expressed full-length L1s using cytoplasmic RNA. With growing repositories of RNA-Seq data, there is potential to mine these datasets to identify and study expressed L1s at single-locus resolution, although many datasets are not strand-specific or not generated from cytoplasmic RNA.

**Results:**

We developed whole-cell, cytoplasmic and nuclear RNA-Seq datasets from 22Rv1 prostate cancer cells to test the influence of different preparations on the quality and effort needed to measure L1 expression. We found that there was minimal data loss in the identification of full-length expressed L1 s using whole cell, strand-specific RNA-Seq data compared to cytoplasmic, strand-specific RNA-Seq data. However, this was only possible with an increased amount of manual curation of the bioinformatics output to eliminate increased background. About half of the data was lost when the sequenced datasets were non-strand specific.

**Conclusions:**

The results of these studies demonstrate that with rigorous manual curation the utilization of stranded RNA-Seq datasets allow identification of expressed L1 loci from either cytoplasmic or whole-cell RNA-Seq datasets.

## Introduction

Mobile elements are repetitive sequences that make up half to two thirds of the human genome [1]. Long interspersed element-1 s (LINE-1 s/L1 s) are the only autonomous, human transposable mobile element [2]. L1 s are able to insert throughout the human genome through an RNA intermediate in an RNA-mediated “copy and paste” mechanism called retrotransposition [3]. They make up 17% of the genome with over 500,000 copies, although only 80–120 L1 elements are thought to be competent for retrotransposition [1, 4]. A full-length L1 RNA able to retrotranspose is about 6 kb in length and must have all the following intact regions: 5′ and 3′ untranslated regions, encoding an internal promoter and associated anti-sense promoter, two non-overlapping open-reading frames (ORFs), and a polyA tail [2, 5, 6]. The L1 ORFs encode a protein with reverse transcriptase and endonuclease activities, and another with RNA-binding and chaperone activities, both of which form an L1 RNP with the L1 mRNA [7–10]. Once this assembly is complete, the L1 RNP reaches genomic DNA and is inserted back into the genome in a process called target primed reverse transcription [11]. It is estimated that a new L1 insertion occurs in the human genome in every 200 births [12]. The expression of these elements has the capacity to contribute to human disease through mechanisms like insertional mutagenesis, target-site deletions, and rearrangements. Over 120 cases of retrotransposition-caused, spontaneous and inherited human diseases have been reported to date. L1 expression and retrotransposition are increased in a variety of epithelial cancers [13–16]. Therefore, there is an urgent need to better understand the spectrum of expressing L1 s, which begins with the accurate identification of authentically expressed, full-length L1 s.

There have been many approaches used to study L1 RNA expression (as reviewed in [17]). Most of them deal primarily with the bulk of mRNA expression of these elements from all L1 loci and a few even make efforts to evaluate the differential expression of the L1 subfamilies [18]. More importantly, most methods do not effectively differentiate between L1 mRNA expression driven by the L1 promoter from the passive presence of L1-related sequences found in other transcripts. Our focus in this method is to differentiate sense transcripts driven by the L1 promoter, which are the only type of L1-related transcripts that are related to the retrotransposition life cycle. Other transcripts, both from the L1 antisense promoter and those incorporated as parts of other RNA species have their own biological roles. However, those latter transcripts only interfere with our understanding of the L1 promoter sense transcripts and are therefore ‘background’ in our studies.

Using RNA Next Generation sequencing (RNA-Seq), we have developed several bioinformatics approaches for locus-specific L1 mRNA expression as previously described [19, 20]. One of these approaches takes a series of steps to filter out the high level (over 99%) of transcriptional noise in RNA-Seq data generated from L1 sequences embedded in other genes whose expression is unrelated to L1 retrotransposition. These steps include selecting for cytoplasmic and polyadenylated transcripts as these full-length L1 RNAs are more likely to be transcribed off their own promoter. We also require that reads align uniquely on the sense strand of L1s, assess expression only from the full-length reference L1s with intact promoters, and finally manually curate each locus to ensure transcription is related to L1 promoter activity [19, 20]. Although this leads to underestimation of the levels of L1 expression and the number of expressed L1 loci, our approach uniquely maps RNA-Seq reads to one locus, which confidently and stringently determine which L1 loci express.

With growing repositories of RNA-Seq data, there is potential to pool and mine these data sets to identify and study expressed L1 s at a single-locus resolution in a variety of models and pathologies [21–23]. However most of these data sets do not come from cytoplasmic RNA samples and many are not strand specific. Here we set out to determine whether the identification of expressed L1 loci using whole cell RNA and/or non-stranded RNA-Seq data could be reliably accomplished. We also set out to determine the extent of data loss in terms of detectable full-length L1 loci expression for each approach compared to the previously published approach [19]. To carry out these studies we generated strand-specific RNA-Seq from 2 biological replicates of the 22Rv1 prostate tumor cell line [24] using whole cell, cytoplasmic, or nuclear preparations. By eliminating strand-specificity from these data, we utilized the same data sets to assess our approach for authentic L1 mRNA expression analysis using non-stranded data sets.

Our findings demonstrate that whole-cell RNA analysis can provide similar results to cytoplasmic L1 RNA analysis. However a close agreement between the two approaches is only possible with rigorous manual curation of the results of whole cell RNA-Seq bioinformatics analysis in order to eliminate high levels of transcripts incorporated as portions of other RNAs (co-transcription). We refer to these co-transcripts as ‘background’ in this manuscript because our focus is on sense transcripts from the L1 promoter and the high levels of L1-chimeric co-transcription interfere with these studies. We also determined that analysis of L1 expression using non-stranded RNA-Seq can identify authentic expression of some L1 loci. However, the number of identified L1 loci is reduced by half as a significant portion of authentic loci cannot be distinguished from the background and a much greater effort in manual curation is required compared to the analysis of stranded cytoplasmic or whole cell RNA-Seq data sets. Our results clearly demonstrate that existing whole cell and/or non-stranded RNA-Seq data sets should not be used for L1 mRNA expression analysis without eliminating every and all sources of background L1 sequences as such analyses produce false positive results.

## Methods

### Prostate tumor cell line, 22Rv1

22Rv1 cells [25] were kindly provided by Dr. Yan Dong. The cells were cultured in RPMI Media 1640 (Life Technologies) supplemented with 10% fetal bovine serum (Life Technologies).

### RNA preparation: whole cell, cytoplasmic, and nuclear

Cells were collected by scraping from two, 75–100% confluent T-75 flasks. The flasks were first washed two times in 5 mL cold PBS (Invitrogen). In the last wash, cells were scraped and transferred to a 15 mL conical tube and centrifuged for 2 min at 1000 rpm at 4 °C and the supernatant was discarded. For whole cell RNA preparations, the cell pellet was added to pre-chilled 7.5 mL Trizol (Invitrogen) and 1.5 mL chloroform (Fisher). For cytoplasmic RNA preparations, the cell pellet was incubated in 500 uL of lysis buffer (150 mM NaCl (Invitrogen), 50 mM HEPES pH 7.4 (Affymetrix), 25 μg/mL digitonin (Research Products International Corp) with 1000 U/mL RNase inhibitor (Invitrogen) added just before use, placed on ice for 5 min and then centrifuged for 2 min at 1000 rpm at 4 °C. The supernatant was added to pre-chilled 7.5 mL Trizol and 1.5 mL chloroform. For nuclear RNA preparations, the pellet remaining after RNA cytoplasmic extraction was added to pre-chilled 7.5 mL Trizol and 1.5 mL chloroform. All Trizol-based solutions were then centrifuged for 35 min at 4000 rpm at 4 °C. The aqueous portion was transferred to 4.5 mL of chilled chloroform and centrifuged for 10 min at 4000 rpm at 4 °C. The resulting aqueous portion was precipitated with 4.5 mL of isopropanol (Fisher) overnight in -80 °C overnight, centrifuged for 45 min at 4 °C at 4000 rpms, washed with 10 mL 100% ethanol (Fisher) and re-suspended in RNAse-free water (Fisher). A further detailed explanation of the RNA preparation is previously described in [20].

### RNA quality check

RNA samples were analyzed for quality on an Agilent 2100 Bioanalyzer System according to the Agilent RNA 6000 Nano kit guide. Cytoplasmic, or whole-cell samples were submitted for sequencing with RIN > 8, and the nuclear RNA sample was submitted without this quality control.

### RNA sequencing

Whole cell, cytoplasmic, and nuclear RNA samples were submitted to BGI genomics for selection of polyadenylated RNAs, and sequencing by the Illumina TruSeq strand-specific, and paired-end library preparation with barcodes. Samples were pooled in groups of 2 and applied to a single lane of an Illumina HiSeq 2500/4000 instrument. Data were sorted based on barcodes attached to each individual sample providing between 150 and 250 million paired-end reads per sample. This represents a higher depth of sequencing than normal in order to provide higher quality data. For detection of L1 locus-specific expression we typically recommend sequencing with around 50 million paired-end reads per sample.

### Annotation for full length L1 s

The annotations for full-length L1s have been previously described [19, 20] and can be found in .gff format in Additional file 1: a-b. Briefly, a Repeat Masker annotation for LINE elements was downloaded from UCSC and intersected with the annotation of a human BLAST search for the first 300 bps of the L1.3 full-length L1 element that encompasses the L1 promoter region [26–28]. The resulting annotation contained about 5000 full-length L1s with intact promoters in the hg19 reference genome used to identify LINE-1 expression at the locus-specific level.

### Bioinformatic analysis

The alignment strategy for RNA-Seq data to the human genome for endogenous L1 expression studies has been previously described [19, 20]. Briefly, in this study we used bowtie1 [24] to map unique transcript reads with the tryhard switch to the human reference genome. Our command requires that the paired ends align concordantly with the human genome and that the software searches exhaustively for the best match and only retains aligned reads that map to one locus better than any other in the genome. Bedtools coverage was used to count mapped reads in a stranded and unstranded manner to all full-length L1s [29]. Bedtools coverage was also used to generate the number of sense reads that mapped upstream the full-length L1s by 1000 and 5000 bps [29].

### Mappability assessment

Our bioinformatic strategy is to only consider reads that mapped uniquely to one locus. In order to better understand and assess how ‘mappable’ regions are in the genome, we downloaded species-specific whole genome Illumina paired-end sequence files from NCBI. We used the same bowtie1 alignment approach as for RNA-Seq to assign whole genome reads that mapped uniquely to the genome [25]. The accession number for a *Homo sapiens* whole genome sequence file used in these studies was ERR492384. A further detailed explanation of how mappability is assessed is previously described in [20].

### Manual Curation of L1 loci

Following the bioinformatic analyses, a table per sample was generated displaying the annotated L1 loci that had 10 or more reads mapping. These full-length L1 loci with mapped RNA-Seq reads were then visually inspected to validate that reads were expressed using the L1 promoter. To manually curate authentically expressed L1s, the gene annotation of the reference genome of interest, the L1 annotation, the RNA-Seq and whole genome alignments were uploaded in IGV, a genomics visualization tool [30]. Any expressed L1 s identified in our bioinformatic pipeline that had sense reads upstream the L1 within 5 kb were rejected as false positives. However, exceptions were developed for this rule. First, if there were minimal reads directly overlapping the L1 promoter start site, but slightly upstream the L1 for 100–200 base pairs, these L1 s were considered to be authentically expressed. Second, any L1s with mapped transcript reads, but with immediately un-mappable upstream regions were curated out as false positives as it could not be confidently determined that expression originated from the promoter region and not upstream transcription. Third, the L1 locus was curated to be a false positive even if there were no sense reads upstream within 5 kb in cases of bordering broad regions of un-annotated expression at similar expression levels to the L1. Finally, if an L1 locus had a pattern of expression un-related to its mappability e.g. a large pile of reads mapped only to the middle of a full-length L1 with complete mappability coverage, then the locus was considered too suspicious to be confidently curated as a L1 expressed using its own promoter. An L1 curated to be a false positive was labeled with a red color and an L1 curated to be authentically expressed was labeled with a green color as seen in Additional file 1:A-E. Whole cell and cytoplasmic RNA from 22Rv1 from replicate 1 were curated together and whole cell, cytoplasmic, and nuclear RNA from 22Rv1 from replicate 2 were curated together. Only L1 loci with a minimum of ten aligned reads were considered for curation unless a locus reached that threshold in one of the other samples in that group. Descriptions of the genomic environment around a curated L1 were noted explaining why each locus was deemed authentically expressed or not. It was also noted if there were any antisense promoter activity.

### Normalization of transcript reads

In order to compare expression at the specific locus level among multiple sequenced samples, the raw transcript reads mapping to each manually curated L1 locus were then normalized by calculating individual L1 loci FPKM values. As the full-length L1s in the human reference genome are all approximately 6 kb in length, the FPKM value was calculated by dividing the number of uniquely mapped transcript reads to an individual L1 locus and the product of the million mapped reads specific to the sequence sample of interest and 6. The described formula is demonstrated here:

$$ FPKM\ of\ L1\  locus\ z=\frac{\# of\ uniquley\ mapped\ reads\ to\ L1\  locus\ z\  in\ sample\ y}{million\ mapped\ reads\ in\ sample\ y\times 6} $$

### Exonic:Intronic measurements

Using the aligned sequence files for each sample as developed in the Bioinformatics Analysis Methods section, the ratio of reads that mapped to the exonic regions over the intronic regions of the following housekeeping genes: B2M, GAPDH, GUSB, HPRT, PGK1, and TK1 were calculated. The average of these ratios for each gene were assessed to give a final exonic:intronic ratio in order to assess the quality of the cytoplasmic/nuclear fractionations. A low ratio for example would indicate more nuclear, pre-processed RNA content in sample.

### Statistical analysis

Data are presented as mean with standard error bars. Data were analyzed by Student’s t-test for *n*  =  2 groups. Statistical analysis was performed using GraphPad Prism.

## Results

### Analysis of stranded, cytoplasmic and whole cell RNA-Seq datasets followed by manual curation leads to detection of a similar subset of expressed L1 loci

**Fig. 1:**
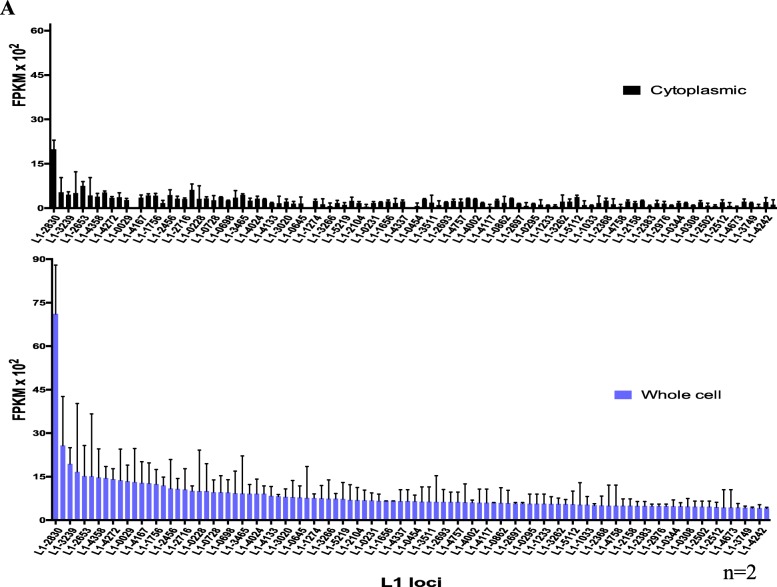
Expressed L1 loci in cytoplasmic versus whole-cell RNA sequencing in 22Rv1 after manual curation. The y-axis denotes the number of uniquely mapped transcript reads as FPKM values × 10^2^. The x-axis denotes the L1 loci identification numbers found to be expressed after manual curation. The same loci are shown in the same order for the cytoplasmic and whole-cell RNA-Seq samples. The bars in black represent averaged normalized reads in the cytoplasmic RNA-seq from the 22Rv1 samples with standard error bars and *n* = 2. The bars in purple represent averaged normalized reads in the whole-cell RNA-Seq 22Rv1 samples with standard error bars and an *n* = 2. Only the first 100 loci ordered from highest to lowest expressing in the whole cell samples are shown out the total 191 loci identified to-be-expressed in order to better fit as many data points as possible and still visualize the distinctive data. The cytoplasmic loci totaled a FPKM of about 3 overall, whie the whole-cell loci expressed at a FPKM close to 10. These numbers are only based on uniquely-mapped reads and are therefore underestimates

**Fig. 2:**
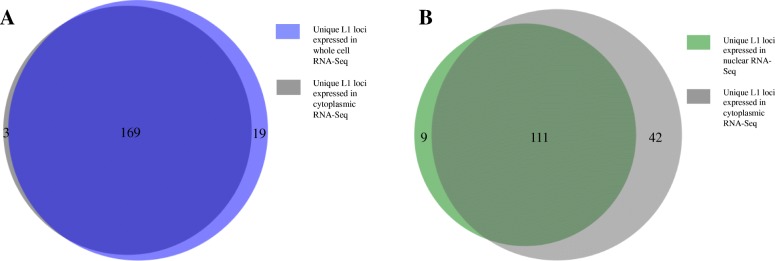
**a** Overlap of expressed L1 loci in cytoplasmic versus whole-cell RNA-Seq of prostate tumor cell line, 22Rv1. The 191 distinct L1 loci identified as expressed after manual curation (Fig. 1) were pooled in the two sets of cytoplasmic and whole-cell extracted RNA and then compared in a proportional Venn diagram [31]. In dark purple are the loci found in both whole-cell and cytoplasmic RNA of 22Rv1. In light purple are the loci found to-be-expressed in only the whole cell RNA-seq preparations of 22Rv1. In grey are the loci found to-be-expressed in only the cytoplasmic RNA-seq preparations of 22Rv1. The number of loci in each shaded region of the diagram is denoted. **b** Overlap of expressed L1 loci in cytoplasmic versus nuclear RNA-Seq of prostate tumor cell line, 22Rv1. The single matching set of cytoplasmic and nuclear extracted RNA were analyzed and manually curated to identify authentically expressed L1 loci. The 162 distinct L1 loci were compared in a proportional Venn diagram [31]. In dark green are the loci found to-be-expressed in both nuclear and cytoplasmic RNA of 22Rv1. In light green are the loci expressed in only the nuclear RNA-Seq of 22Rv1. In grey are the loci found in only the cytoplasmic RNA-Seq of 22Rv1. The number of loci in each shaded region of the diagram is denoted

In order to compare L1 expression using whole cell vs. cytoplasmic RNA preparations, two replicates of each were poly-A selected and sequenced using a strand-specific protocol. The sequencing reads were mapped to the human reference genome using an alignment strategy that looks exhaustively for concordant matches to each read pair throughout the genome and selects only those that map to one location better than any other. The number of sense reads mapping to specific full-length L1 loci were extracted and sorted by read counts. L1 loci and their corresponding reads were then manually curated as described in the Methods and as previously reported [20]. Examples of L1 loci that were curated to be authentically expressed and those that were curated out to have transcription un-related to the L1 promoter are shown in Additional file 6: Figure S1A-D. The manually curated datasets for each of the strand-specific sequencing samples with labeled information like chromosome location by L1 ID and subfamily are found in Additional file 1: A-E. After the curation that identified L1s expressed from their own promoter, reads uniquely mapped to these L1 loci were normalized to FPKM values per specific L1 locus in each sample (Additional file 1**)**. Overall the majority of loci identified to be authentically expressed were found in both replicates of the cytoplasmic- and whole cell- 22Rv1 RNA samples **(**Fig. 1**)** indicating that the two RNA preparations yield similar results when used to identify expressed L1s. Specifically, there were a total of 191 distinct loci identified to be authentically expressed in the cytoplasmic and whole cell RNA sequenced data in which 169 loci were found in both preparations, 3 uniquely found in cytoplasmic preparations, and 19 uniquely found in the whole cell preparations (Fig. 2a). Most of these L1 loci that are uniquely found in the cytoplasm or whole cell RNA preparations are expressed at very low levels and fall below our analysis thresholds in the differently prepared samples. Likely these poorly expressed L1 s would otherwise not be detected when using 50 M read sequencing depth instead of the 150-200 M read sequencing depth. It was observed that there was more relative expression found in the whole-cell L1 loci compared to cytoplasmic L1 loci **(**Fig. 1**)**. When the expressed L1 s were subdivided by subfamily compared to all the full length L1s in the genome, there was a nearly two-fold enrichment for L1PA2 and L1PA3 L1s and a greater than 2 fold decrease for L1PA4 and L1PA5 L1s (Additional file 7: Figure S2A-C). The percentage of expressed L1HS L1s approximately matched the percentage of annotated L1HS L1 s in the human genome (Additional file 7: Figure S2A-C). These data support that our detection method is more sensitive for older L1PA2 and L1PA3 elements because of more identifiable variation among them.

In order to understand the trending difference in levels of L1 expression between cytoplasmic RNA and whole cell RNA samples, matched cytoplasmic and nuclear RNA preparations were generated, sequenced, and analyzed as described in the Methods. After manual curation of L1 loci identified in these stranded cytoplasmic and nuclear 22Rv1 samples (Additional file 1: D-E), the total L1 expression levels were normalized to the sequencing depth of each sample. (Additional file 2). Between these two samples, there were a total of 162 distinct loci identified as authentically expressed L1 loci. Among these 162 loci, 111 loci were found in both preparations, 42 were uniquely found in cytoplasmic preparations, and 9 were uniquely found in the whole-cell preparations (Fig. 2a). The high number of uniquely found loci in the cytoplasmic RNA data is more likely a reflection of the large relative amount of intronic reads in the nucleus that produce transcriptional background noise unrelated to L1 mRNA expression from its promoter which interferes with the ability to confidently call expressed L1s according to our manual curation guidelines described in the Methods (Fig. 4) and as previously reported [20]. Most likely the total L1 expression in the stranded nuclear data is therefore underestimated. These data demonstrate that the two cellular compartments both contain L1 mRNA transcripts and provide an explanation as to why there is more L1 expression in the whole-cell prepared RNA sequenced data compared to the cytoplasmic RNA sequencing data.

### There is less transcriptional background noise related to L1 expression in cytoplasmic versus whole-cell RNA-Seq samples and therefore the former require less manual curation

**Fig. 3:**
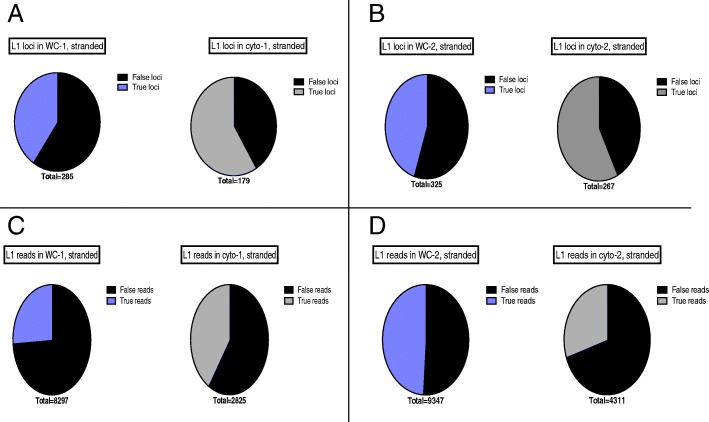
**a**-**b** Curation required by number of L1 loci in strand-specific cytoplasmic and whole-cell RNA-Seq data from replicates 1 and 2. Depicted are pie charts of the number of L1 loci that were curated to be truly or falsely expressed in strand-separated RNA-Seq data from whole cells and cytoplasm. In black are the false loci, in grey are the true loci identified in cytoplasmic RNA samples, and in purple are the true loci identified in whole-cell RNA samples. The number of total curated L1s is denoted beneath the pie charts. **c**-**d** Curation required by number of mapped reads to L1 loci in stranded cytoplasmic and whole cell RNA-seq data from replicates 1 and 2. Depicted are pie charts of the number of sense-oriented reads mapping to L1 loci that were curated to be truly or falsely expressed in strand-separated RNA-Seq data from whole cells and cytoplasm. In black are the false reads, in grey are the true reads identified in cytoplasmic RNA samples, and in purple are the true reads identified in whole cell samples. The number of total curated reads is denoted beneath the pie charts

Cytoplasmic, strand-specific polyA RNA-Seq data provide the best overall analysis of authentic L1 loci expression because active L1 mRNAs must be translated in the cytoplasm. Any RNA that does not reach the cytoplasm cannot participate in the L1 life cycle. We found that the whole-cell RNA-Seq data provided a similar picture of L1 RNA expression to that seen in the cytoplasm, but required substantially more manual curation (Additional file 1), presumably to remove higher background levels from the nucleus. In the 1st replicate of cytoplasmic 22Rv1 RNA sequenced in a strand-specific manner, 179 loci with 2825 reads had to be manually curated with 60.3% loci and 42.2% reads found to be authentically expressed. In comparison, in the 1st replicate of whole-cell prepared 22Rv1 RNA sequenced in a strand-specific manner, 285 loci or 8296 reads had to be manually curated with 40% loci and 27.2% reads found to be authentically expressed (Fig. 3a, c). In the 2nd replicate of cytoplasmic 22Rv1 RNA sequenced in a strand-specific manner, 267 loci or 4311 reads had to be manually curated with 57.3% loci and 30.1% reads found to be authentically expressed. In the 2nd replicate of whole cell prepared 22Rv1 RNA sequenced in a stranded manner, 325 loci or 9347 reads had to be manually curated with 55.1% loci and 49.0% reads found to be authentically expressed (Fig. 3b, d). These metrics are also articulated in Table 1 for further clarification. Overall these data demonstrate that more manual curation is required in order to identify authentically expressed L1s in whole-cell compared to cytoplasmic RNA-Seq data.

**Table 1:**
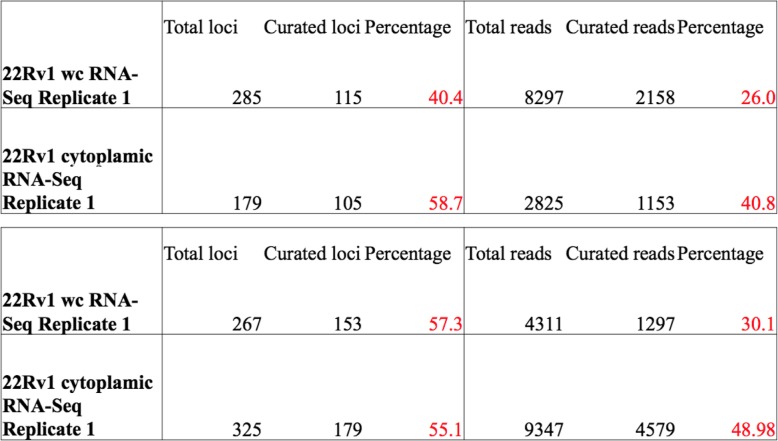
Metrics on the curation required in stranded cytoplasmic and whole-cell RNA-seq data from replicates 1 and 2

In order to verify quality of the cytoplasmic fractionation, the ratio of exonic reads to intronic reads was determined in each of the samples as described in the Methods with raw data shown in Additional file 3:A-E. The exonic to intronic ratio was determined for every sequenced sample by dividing and then averaging exonic and intronic reads that mapped to housekeeping genes. In replicate 1, the exon to intron ratio is 21.7 and 9.4 in the cytoplasmic and the whole-cell RNA samples, respectively (Additional file 8: Figure S3). In replicate 2, the exon to intron ratio is 18.2 and 14.9 in the cytoplasmic and the whole-cell RNA samples, respectively (Additional file 8: Figure S3). The smaller difference in exon:intron ratios in replicate 2 compared to replicate 1 can explain why there is less of a difference in the amount of manual curation required for cytoplasmic versus whole cell prepared RNA-Seq samples in replicate 2 compared to replicate 1 (Fig. 3).

**Fig. 4:**
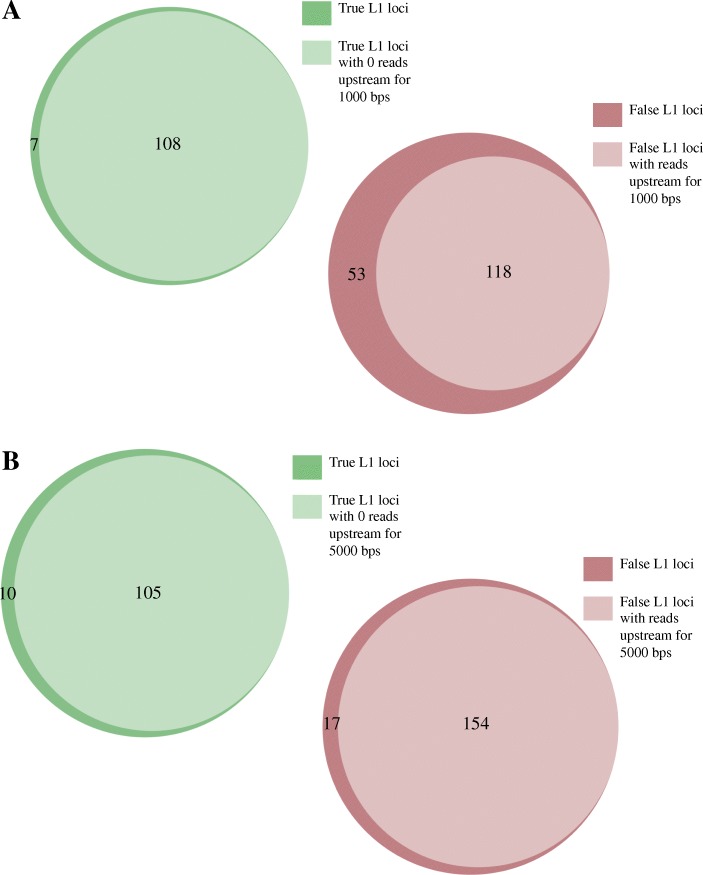
Partial automation of the curation process. **a** Number of L1 loci with mapped reads upstream by 1000 bps in the same orientation from replicate 1, strand-specific, whole-cell RNA. **b** Number of loci with mapped reads upstream by 5000 bps in the same orientation from replicate 1, strand-specific, whole-cell RNA. The total 285 L1 loci identified to have uniquely mapped reads in the sense orientation to full-length L1 s in the human reference genome in replicate 1, whole-cell RNA-Seq data of 22Rv1 were separated by loci curated to be consistent with expression from the L1 promoter (true) and loci falsely expressed from a different promoter and then compared to regions of upstream, sense expression in a proportional Venn diagram [31]. In light green are the L1 loci identified to be authentically expressed after manual curation in which there were zero mapped reads upstream in the same direction for up to 1 or 5 kb upstream. In dark green are the L1 loci identified to be authentically expressed after manual curation in which there were a few mapped reads upstream in the same direction for up to 1 or 5 kb upstream. In light red are the L1 loci identified to have expression unrelated to L1 promoter transcription after manual curation in which there were mapped reads upstream in the same direction for up to 1 or 5 kb upstream. In dark red are the L1 loci identified to have expression unrelated to L1 promoter transcription after manual curation in which there were not mapped reads upstream in the same direction for up to 1 or 5 kb upstream. The numbers of L1 loci in each group are denoted within the Venn diagrams

Because there is more manual curation required to identify expressed L1 s in strand-specific whole-cell RNA-Seq samples, we began to identify rules that distinguish authentically expressed L1 loci from the background and therefore can be applied to automate our manual curation process as is described in the Methods. Using bedtools coverage, we extracted the number of sense-oriented reads identified either 1000 or 5000 bps upstream of all full-length L1s in the human reference genome [29]. We used the whole-cell RNA-Seq from replicate 1 for this analysis. 108 out of 115 authentically expressed L1s had zero reads in the 1000 bps upstream region (Fig. 4a). Of the L1s determined to have transcription unrelated to the L1 promoter in this sample, 118 out of 171 had reads in the upstream 1000 base pairs (Fig. 4a). If all L1 loci with upstream sense reads up to 1000 base pairs were filtered out, about 10% of the authentic L1 loci would be lost. With this automation approach, the number of loci requiring curation would be reduced by 41%. When we expand the region upstream of the L1 s to 5000 bps, 105 out of 115 authentically expressed L1 s had zero sense, upstream reads (Fig. 4b). Of the authentic L1 expressed loci in whole-cell RNA-Seq from replicate 1, 154 out of 171 loci had reads upstream up to 5000 base pairs (Fig. 4a). Using 5000 base pairs upstream to automatically curate the L1 loci, about 10% of the L1 loci determined from manual curation would be lost, but the number of loci requiring curation would be reduced by about 54% (Additional file 9: Figure S4A). Next steps include further refining automation by taking into consideration the ratio of L1 mapped reads to upstream mapped reads and other features such as upstream expressed exons, but we have yet to fully determine how mappability of both the elements and their flanking sequences should be handled.

### Analysis of non-strand-specific RNA sequencing data requires twice as much manual curation as strand-specific data and results in the loss of half of the authentic expressed L1 loci

**Fig. 5:**
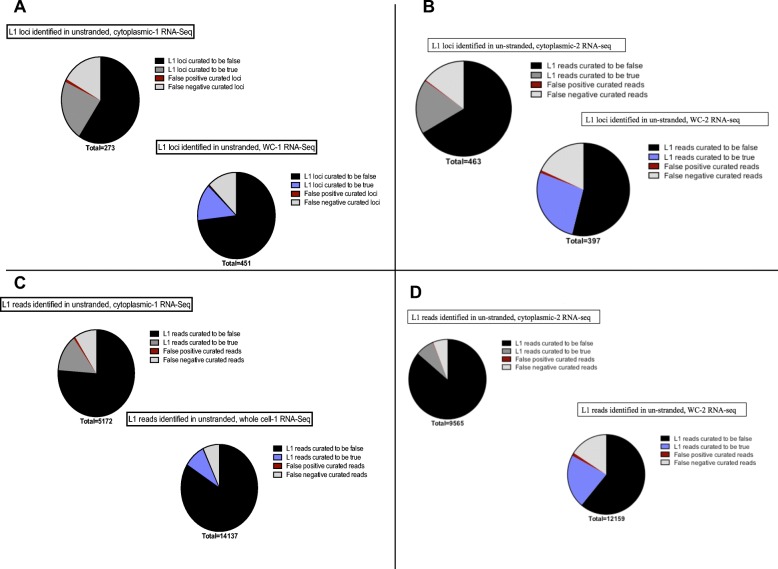
Curation required for data that is not strand specific. **a**-**b** Curation required by number of L1 loci in un-stranded cytoplasmic and whole cell RNA-seq data from replicates 1 and 2. Depicted are pie charts of the number of L1 loci that were curated to be truly or falsely expressed in non-strand-specific RNA-Seq data from whole cells or the cytoplasm. These curations were then compared to manual curation results of the matched strand-specific data in order to determine false positive and false negative calls. In black are the curated-to-be false loci, in light grey are the false negative calls determined when compared to strand-specific data, in red are the false positive calls made when compared to strand-specific data, in dark grey are the true loci identified in cytoplasmic RNA samples, and in purple are the true loci identified in whole-cell RNA samples. The number of total curated L1 loci is denoted beneath the pie charts. **c**-**d** Curation required by number of mapped reads to L1 loci in stranded cytoplasmic and whole cell RNA-seq data from replicates 1 and 2. Depicted are pie charts of the number of L1 mapped reads that were curated to be truly or falsely expressed in non-strand-specific RNA-seq data whole cells and cytoplasm. These curations were then compared to manual curation results of the matched strand-specific data in order to determine false positive and false negative calls. In black are the false reads, in light grey are the false negative calls determined when compared to strand-specific data, in red are the false positive calls made when compared to strand-specific data, in dark grey are the true L1 reads identified in cytoplasmic RNA samples, and in purple are the true L1 reads identified in whole cell RNA samples. The number of total curated L1 s reads is denoted beneath the pie charts

Because many available RNA-Seq datasets are not strand-specific, we wished to determine if they can be used for reliable detection of locus-specific L1 mRNA expression. We utilized the RNA-Seq data from both cytoplasm and whole-cell RNA from 22Rv1 samples described above, but ignored the strand specificity. The manually curated data sets for the two replicates of whole cell and cytoplasmic RNA sequenced samples in a non-stranded manner are found in Additional file 5: A-D. In the 1st replicate of cytoplasmic RNA extracted from 22Rv1 cells analyzed in the non-stranded manner, 273 loci or 5172 reads had to be manually curated. Sixty-two loci and 712 reads were found to be authentically expressed and 162 loci or 3940 reads identified to be falsely expressed **(**Fig. 5a, c**)**. Three loci with a total of 40 reads were curated as authentically expressed in the non-stranded data when in fact the mapped reads were antisense to the L1. These loci became false positive calls when the non-stranded format was used **(**Fig. 5a, c**)**. There were 46 loci or 480 reads that were curated to have expression un-related to the L1 promoter because of non-stranded upstream reads which were labeled as false negative calls **(**Fig. 5a, c**)**. These L1 loci were authentic L1s according to the analysis of the matched strand-specific data, but had antisense promoter related upstream reads. In the 1st replicate of whole cell prepared 22Rv1 RNA analyzed in a non-stranded manner, 451 loci or 14,137 reads had to be manually curated. Sixty-three loci and 712 reads were found to be authentically expressed and 330 loci and 11,863 reads found to be falsely expressed **(**Fig. 5a, c**)**. Two loci with a total of 20 reads were curated to be expressed in the non-strand-specific data when the mapped reads were antisense to the L1 so were marked as false positive calls **(**Fig. 5a, c**)**. There were 56 loci or 986 reads that were curated to have expression un-related to the L1 because of non-stranded upstream reads, but were authentic L1s according to the matched strand-specific data with antisense promoter-related upstream reads. These were consequently labeled as false negative calls **(**Fig. 5a, c**)**. The second biological replicate of whole cell and cytoplasmic, non-strand-specific RNA-Seq followed the same pattern and distribution of true loci, false loci, false positive loci, and false negative loci when compared to their matched stranded data sets (Fig. 5b, d). These metrics are also articulated in Table 2 for further clarification. Interestingly, we observed a number of instances of mappable, full-length L1s with no sense expression within the L1 could have patterns of expression consistent with antisense promoter activity indicating that the sense and antisense promoters of L1 can be uncoupled (Additional file 5, Additional file 9: Figure S4). Overall these data demonstrate that analysis of the non-strand-specific sequencing data doubles the amount of required manual curation and cuts the number of identified, authentically expressed L1 in half.

**Table 2:**
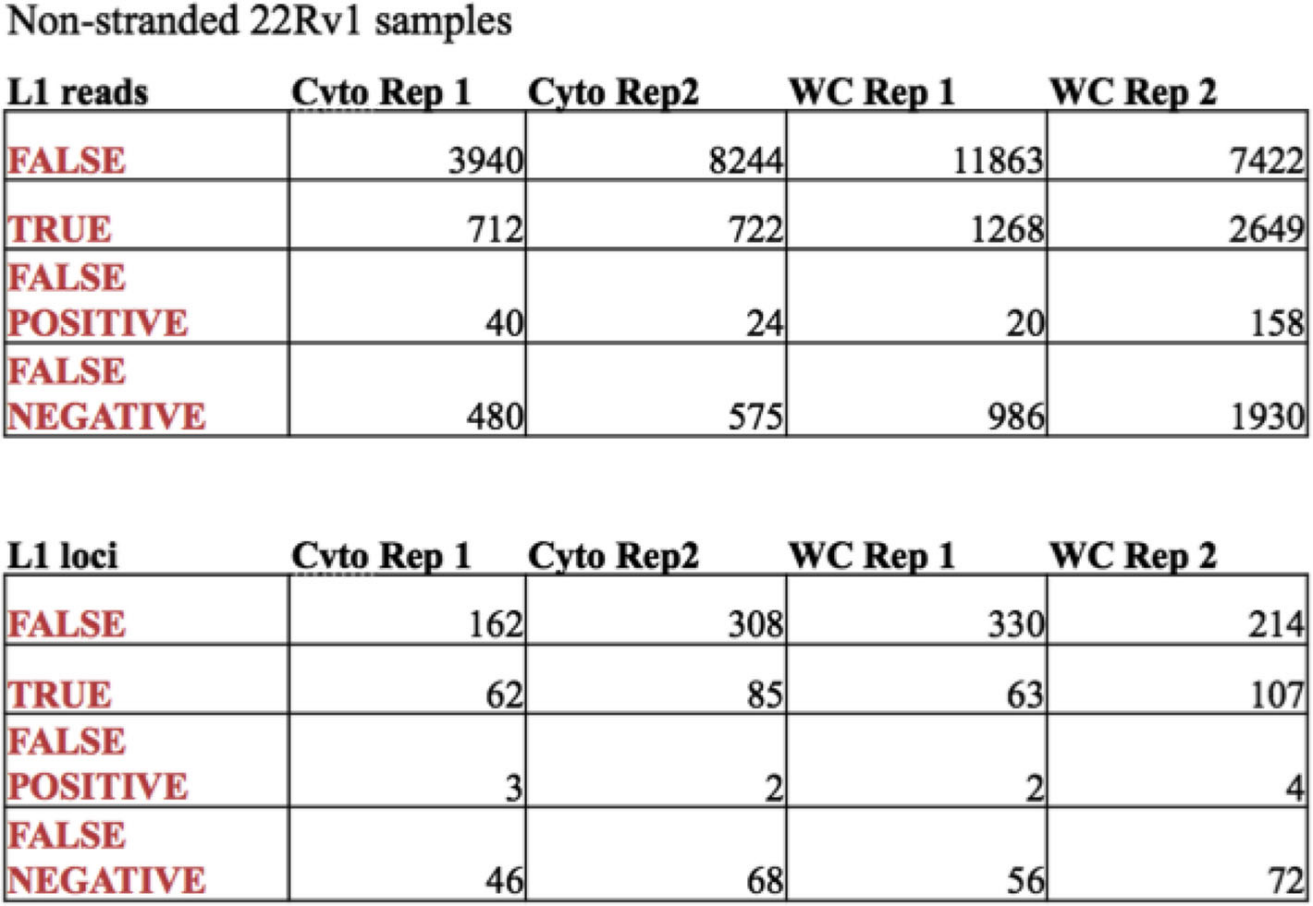
Metrics on the curation required in non-stranded cytoplasmic and whole-cell RNA-seq data from replicates 1 and 2

## Discussion/conclusion

L1 activity is known to cause new genetic diseases through insertional mutagenesis, the creation of double-stranded breaks, and the induction of non-allelic homologous recombination [12, 32, 33]. Studies of L1 mobilization have been limited because of the difficulties created by hundreds of thousands of defective copies when only a few copies are able to actively undergo retrotransposition [4]. The limited number of active elements is at least partly due to only a small subset transcribing in any given cell type [19, 34]. Utilizing Next Generation RNA-Seq, we have developed an approach mapping RNA transcripts to full length L1 s annotated in the human genome in order to reliably identify expressed L1 s at the locus-specific level [16]. This approach selects only for those reads that align to one locus better than any other and are contiguous with the genome as would be expected for a L1 transcript. However, we also utilize strand-specific RNA-Seq from cytoplasmic, polyadenylated RNA to help eliminate background from elements passively incorporated into other RNA molecules. Even with these steps, the most labor intensive part of the procedure is a manual curation to validate that the read alignments are consistent with expectations for transcription from the L1 promoter.

Many existing RNA-Seq datasets are either not strand-specific or do not utilize cytoplasmic RNA. Therefore, we have explored the importance of these variables on this mapping strategy. Of particular interest, we found that whole-cell RNA-Seq studies could generate almost the same quality of data as cytoplasmic RNA-Seq, but require about twice as much manual curation. This makes available the use of strand-specific whole-cell datasets, as well as new experiments in situations where isolation of cytoplasmic RNA would be difficult. These might include some studies from stored cells or tissues where the nucleus may not remain intact. In contrast, experiments that were not carried out with strand-specific RNA-Seq required even more manual curation. These non-stranded RNA-Seq studies were also unable to detect about half of the expressed L1 loci (Fig. 5). Thus, although non-strand-specific datasets can provide some preliminary assessment of L1 expression, it will be much more limited. A good example of one reason these problems occur is that authentic L1 antisense transcripts cannot be distinguished from sense transcripts when strand-specific information is missing.

Younger and more active L1 elements have had less time to accumulate differences from one another so this unique-mapping approach underestimates the number of these expressed L1 s. Our approach also underestimates the quantity of L1 expression by not considering reads that map equally to more than one genomic location. One way to recover some of the expressed, unmappable L1s from RNA-Seq data is to identify whether they have transcripts that extend into downstream flanking sequences [19, 34]. This problem can also be partly solved using long 5′ RACE techniques and long sequencing to get better discrimination with the mapping of the transcripts [19]. The quantitation of L1 mRNA expression can also be better adjusted by correcting for the relative mappability of the different L1 loci [20]. Despite the difficulties mapping to the younger and more active L1 elements, we were able to detect expression from one of the hot L1 elements in the human genome [4] that retained over 10% L1 activity relative to a strong reference L1. This element, FL-L1–5219, one of multiple full-length elements in the TTC28 gene has been shown to be the most insertionally active locus in a prostate cancer cell line [14] and is also active in several other cancers. Thus, combining measurements of the retrotransposition capability of individual loci with measurements of their expression is consistent with the observed retrotransposition rates.

There are recently made available bioinformatic tools that work to quantify TE transcript abundances by assigning multi-mapped reads proportionally to TEs like TEtranscripts and TESalmon [35, 36]. Another method to compare differences in L1 expression quantitation at the locus specific level includes using iterative improvements in assigned fractions of multi-mapping reads as seen with the SQuIRE bioinformatics pipeline [37]. SQuIRE demonstrates that the locus-specific transcripts it maps are from different types of transcripts, but stops short of separating them according to whether they come from the L1 promoter or not. More recently, L1EM takes a novel approach to separate what they term ‘passive’ transcripts from those that arise from the L1 promoter [38]. Their approach appears to be robust in cells with higher levels of authentic L1 mRNA expression. However, they note that there is little or no L1 mRNA expression in most normal tissues. At these low levels of L1 mRNA expression, manual curation is still the most reliable approach. Their study also agrees with our finding that non-stranded RNA-Seq greatly decreases reliability of detection of L1 mRNA expression. SQuIRE and L1EM both use methods to assign multi-mapped reads to specific loci in order to improve quantitation. All of these approaches, including ours, have limited sensitivity in detecting polymorphic L1s even though there is evidence that they are highly expressed [34, 39]. In order to detect these polymorphic elements the first next steps include construction and insertion of polymorphic sequences into the reference genome. This approach was used successfully to detect expression from the subset of polymorphic L1HS elements whose transcripts readthrough the L1 polyA site into downstream flanking sequences (34).

Manual curation is the rate-limiting step in our protocol and significantly limits the volume of studies that can be carried out. The primary factor indicating passive L1 inclusion in another transcript is the presence of upstream reads suggesting that there is a different promoter somewhere upstream of the L1. Therefore, we performed studies to determine whether we could automate the major issue causing the need for manual curation, i.e. non-specific transcription through a L1 element. We found that with modest loss of data (10% of authentically identified, expressed L1 loci), the amount of manual curation could be cut approximately in half (Fig. 5). Ultimately, the usefulness of carrying out studies with any of the approaches that miss extensive L1 transcripts or significantly increase background will depend on the goals of the study. For clean and comprehensive data in regards to L1 expression, the transcriptional background noise must be considered and properly handled. It is also important to note that our studies were carried out in a cancer cell line with moderately high L1 expression. In cells with much lower L1 expression, the importance of manual curation becomes even greater as it is difficult to assess the level of authentic L1 expression prior to carrying out the full analysis.

Although our goal has been primarily an understanding of expression of the L1 elements themselves, it is worth noting that including antisense reads in our analysis allowed us to find transcripts from the antisense L1 promoter as well. While it is known that the sense L1 promoter can make transcripts without the formation of stable antisense transcripts [6], our findings show that L1 ASP activity could be uncoupled from the L1 sense promoter and form stable antisense transcripts from a L1 locus without any apparent sense transcription. This agrees with the findings of another previously published study [34]. This provides the potential for the ASP to alter expression of nearby genes and provide antisense L1 transcripts that could in turn alter *in trans* expression of sense L1 transcripts generated by other L1 loci [40]. Another biological point of note is that we found that there were extensive levels of L1 transcripts in the nucleus as well as the cytoplasm (Fig. 1). The nuclear transcripts were very similar to the ones found in the cytoplasm and it seems likely that their relative abundance suggests that either L1 RNAs do not escape the nucleus completely, are slower to transport than mature mRNA species, or are awaiting re-integration into the genome.

Although we still recommend cytoplasmic mRNA for studying L1 expression, this study provides strong support that with rigorous curation high quality data can be obtained from whole cell RNA preparations. Strand-specific RNA-Seq seems to be the most important criteria in obtaining high quality mapping data for L1 loci. With growing repositories of RNA-Seq samples available to study, it is critical that we are able to maximize the impact of these data on our understanding of mobile element biology.

## Supplementary information

**Additional file 1.** Manually curated set of L1 s with uniquely mapped sense reads in stramd-specific 22Rv1 A) whole cell RNA-Seq data from replicate 1, B) cytoplasmic RNA-Seq data from replicate 1, C) whole cell RNA-Seq data from replicate 2, D) cytoplasmic RNA-Seq data from replicate 2, and E) nuclear RNA-Seq data from replicate 2. L1 s curated to be authentically expressed were labeled with a green color and L1 s curated to be rejected as authentically expressed were labeled with a red color and its reason for rejection or acceptance was noted in the most right column.

**Additional file 2.** FPKM values for manually curated true L1 expression.

**Additional file 3.** Exon:Intron ratio calculations: A) whole-cell RNA-Seq data from replicate 1, B) cytoplasmic RNA-Seq data from replicate 1, C) whole-cell RNA-Seq data from replicate 2, D) cytoplasmic RNA-Seq data from replicate 2, and E) nuclear RNA-Seq data from replicate 2. The number of mapped reads from the RNA-Seq sample of interest to the exons and introns of different housekeeping genes like B2M, GAPDH, GUSB, HPRT, PGK1, TK1 are in column J. The sum of exon reads and intron reads for each gene are in column N. The ratio of exon:intron calculations are in column O. The average of these ratios per Seq sample are found in column O, row 98.

**Additional file 4.** The number of uniquely mapped upstream reads up to 1000 and 5000 bps upstream aligned with the manually curated, strand-specific, whole-cell, RNA-Seq data from replicate 1. In the first column are the L1 locus ID numbers, in the second column are the number of sense reads that map uniquely to the specific L1, in the third column is the reason for acceptance or rejection as authentically expressed L1 s, in the fourth column are the number of sense reads uniquely mapping up to 1000 bps upstream the specific L1, and in the fifth column are the number of sense reads uniquely mapping up to 5000 bps upstream the specific L1. In green are the L1 s curated to be expressed off their own promoters. In red are the L1 s curated to be passively transcribed off a promoter unrelated to the L1.

**Additional file 5.** Manually curated set of L1 s with uniquely mapped non-strand-specific reads in 22Rv1 stranded in whole cell RNA-seq data from replicate 1. L1 s curated to be authentically expressed were labeled with a green color and L1 s curated to be rejected as passively expressed were labeled with a red color and its reason for rejection or acceptance was noted in the most right column following the guidelines for manual curation. In purple are examples of L1 s with antisense promoter activity. As the orientation of reads can not be distinguished in non-stranded data these L1 loci were curated to be not expressed off their own promoter and represent false negative calls. In blue are L1 loci that were curated to be authentically expressed in non-stranded data, but in fact had antisense reads mapped to it. These were considered false positive calls.

**Additional file 6: Figure S1.** Examples of curated L1 loci in 22RvI. Loaded into IGV are the human reference genome, the human full-length L1 annotation, whole cell 22RvI bam file from replicate 1, and lastly the genomic HeLa bam file to assess mappability, which are all available upon author request. Arrows have been added to aid in the visualization of direction of the annotated L1. Arrows and reads in red are oriented in sequence from right to left. Arrows and reads in blue are oriented in sequence from left to right. A) In IGV, this L1 locus appears to be expressed off its own promoter as there are no reads upstream the L1 in the sense orientation for over 5 kb. This L1 has low mappability and is within a gene of opposite direction. B) In IGV, this L1 locus was rejected as an expressed L1 as there are upstream reads in the same orientation within 5 kb. This L1 is within a gene of the same direction so the transcript reads are most likely originating from the promoter of the expressed gene. C) In IGV, this L1 locus was rejected as an expressed L1 as there are upstream reads in the same orientation within 5 kb. This L1 is downstream of a highly expressed gene in the same direction so the transcript reads are most likely originating from the promoter of that expressed gene and extending beyond the normal gene terminator. D) In IGV, this L1 locus was rejected as an expressed L1 as there are upstream reads in the same orientation within 5 kb. This L1 is not within or near an annotated gene in the reference gene so the origin of these transcripts within and upstream of the L1 element suggest an un-annotated promoter.

**Additional file 7: Figure S2.** A) Subfamily distribution of full length L1 s in the human genome. B) Subfamily distribution of full length L1 s expressed in the whole cell preparation of 22Rv1 with *n* = 2. C) Subfamily distribution of full length L1 s expressed in the cytoplasmic preparation of 22Rv1 with *n* = 2. Colors are designated according to the legend by subfamilies L1HS, L1PA2, L1PA3, L1PA4, L1PA5, L1PA6, L1PA7, L1PA8, and Other. The other category includes L1MA4A, L1MA7, L1P1, L1P2, L1PA16, L1PA8A, L1 PB1, and L1BP4. Percentages of the L1 subfamilies are noted around the pie charts.

**Additional file 8: Figure S3.** A) Estimated ratio of exonic reads to intronic reads in replicate 22Rv1 RNA-seq samples. The black bars represent the ratio of exonic to intronic reads in the cytoplasmic RNA samples, the purple bars represent the ratio of exonic to intronic reads in the whole cell RNA samples, and the green bar represents the ratio of exonic to intronic reads in the nuclear RNA samples.

**Additional file 9: Figure S4.** Example of L1 with antisense promoter activity de-coupled from sense promoter activity visualized in IGV. Loaded into IGV are the human reference genome, the human full-length L1 annotation, WC 22RvI bam file from replicate 1, and lastly the genomic HeLa bam file to assess mappability, which are all available upon author request. Arrows have been added to aid in the visualization of direction of the annotated L1. Arrows and reads in red are oriented in sequence from right to left. Arrows and reads in blue are oriented in sequence from left to right.

## Data Availability

The datasets used and/or analyzed during the current study including the original fastq sequencing files are available from the SRA database under the accession numbers SAMN11831215, SAMN11831216, SAMN11831217, SAMN11831218, SAMN11831219. All annotations files and generated output data sets corresponding to number of reads mapping housekeeping genes to L1 s with manual curation are included in this published article (and its supplementary information files).
