# Supplementary figures and images for: Comparative analysis on the expression of L1 loci using various RNA-Seq preparations
Source: Mob DNA. 2020 Jan 6;11:2. doi: 10.1186/s13100-019-0194-z (PMC6945437; doi:10.1186/s13100-019-0194-z)

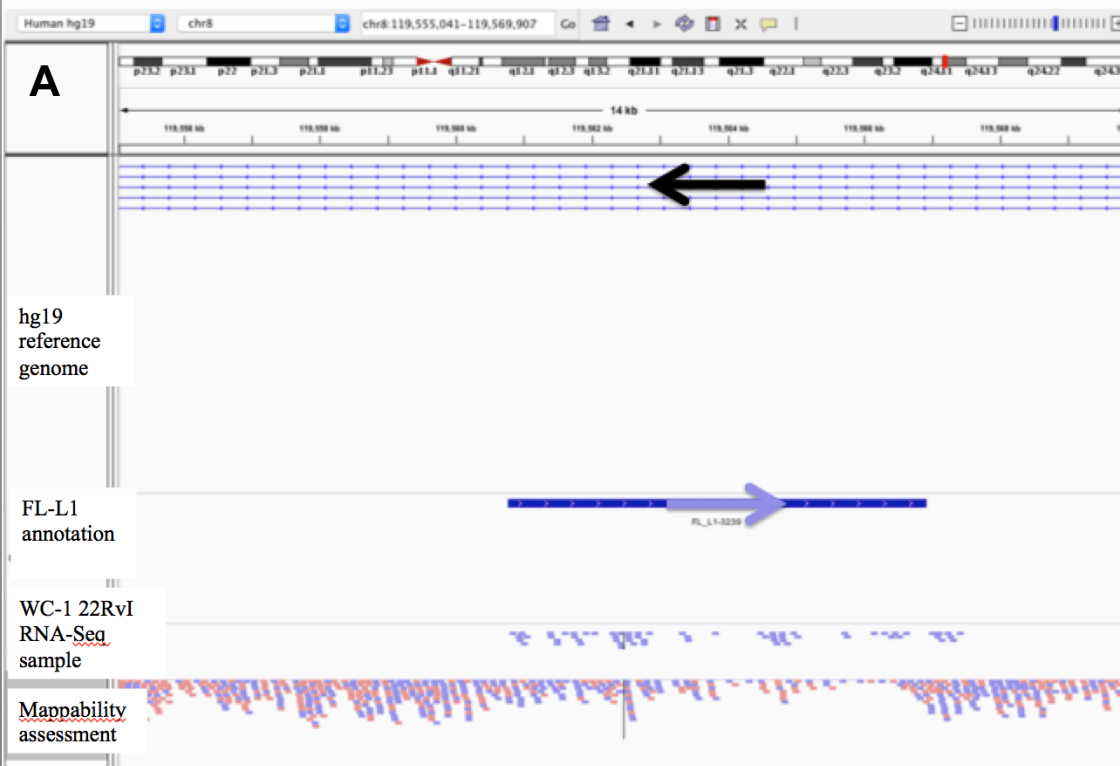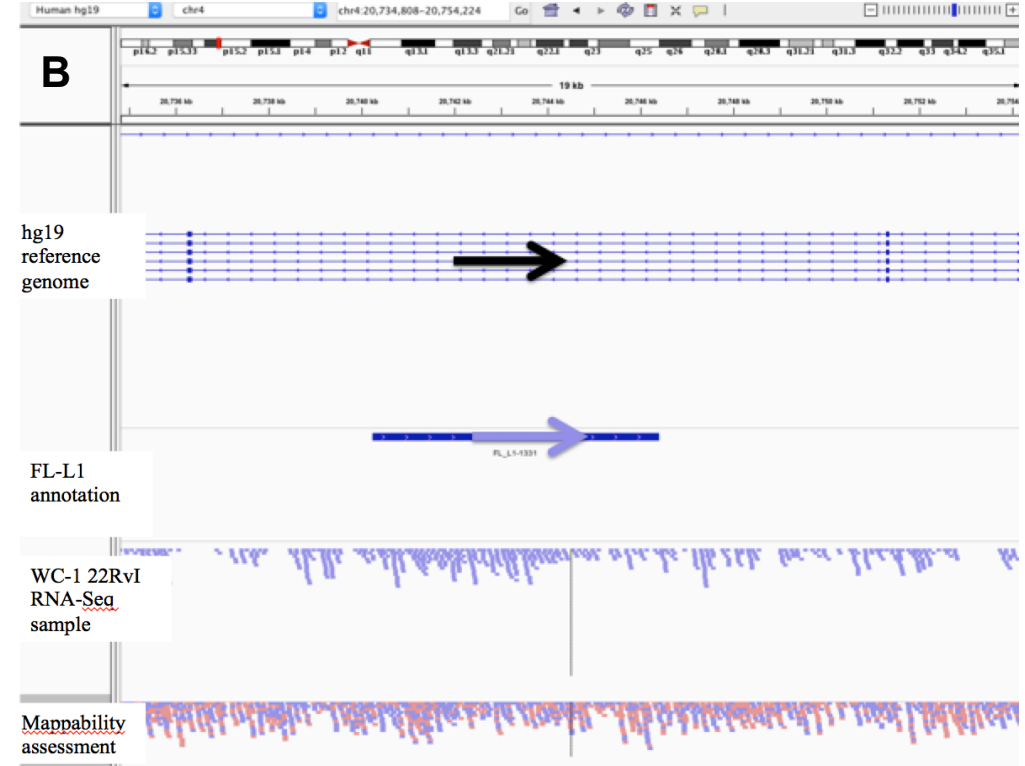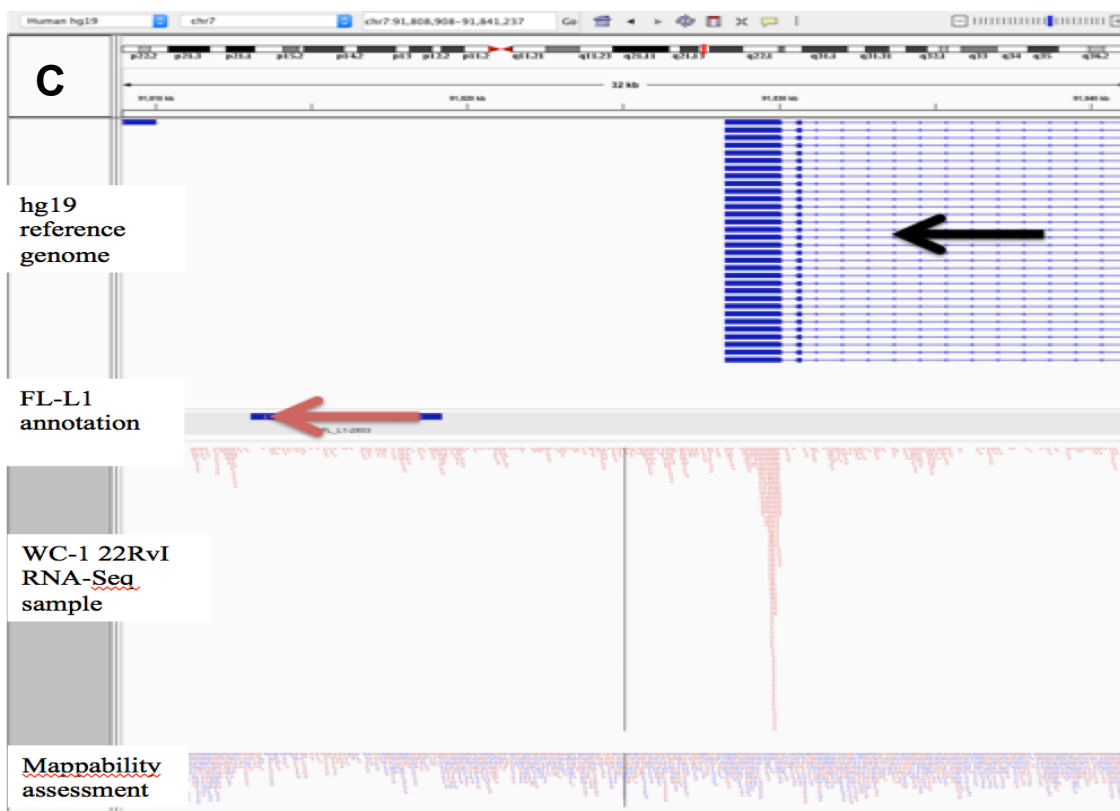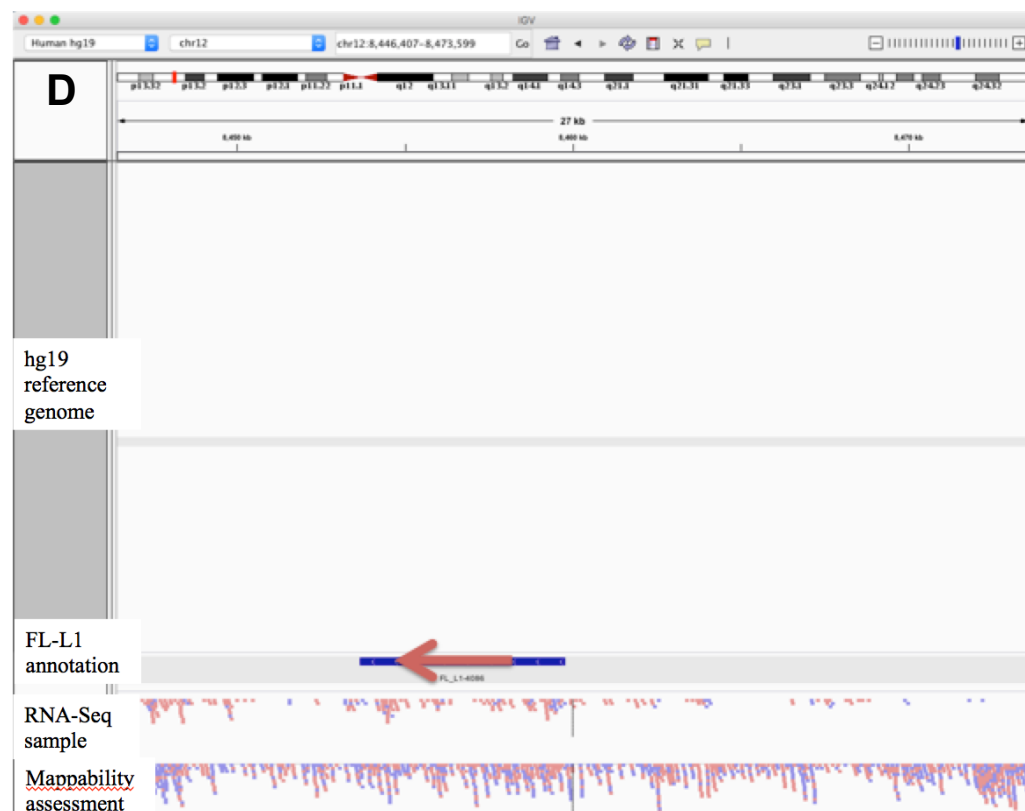

Supplement: Supplementary file 6 — Additional file 6: Figure S1. Examples of curated L1 loci in 22RvI. Loaded into IGV are the human reference genome, the human full-length L1 annotation, whole cell 22RvI bam file from replicate 1, and lastly the genomic HeLa bam file to assess mappability, which are all available upon author request. Arrows have been added to aid in the visualization of direction of the annotated L1. Arrows and reads in red are oriented in sequence from right to left. Arrows and reads in blue are oriented in sequence from left to right. A) In IGV, this L1 locus appears to be expressed off its own promoter as there are no reads upstream the L1 in the sense orientation for over 5 kb. This L1 has low mappability and is within a gene of opposite direction. B) In IGV, this L1 locus was rejected as an expressed L1 as there are upstream reads in the same orientation within 5 kb. This L1 is within a gene of the same direction so the transcript reads are most likely originating from the promoter of the expressed gene. C) In IGV, this L1 locus was rejected as an expressed L1 as there are upstream reads in the same orientation within 5 kb. This L1 is downstream of a highly expressed gene in the same direction so the transcript reads are most likely originating from the promoter of that expressed gene and extending beyond the normal gene terminator. D) In IGV, this L1 locus was rejected as an expressed L1 as there are upstream reads in the same orientation within 5 kb. This L1 is not within or near an annotated gene in the reference gene so the origin of these transcripts within and upstream of the L1 element suggest an un-annotated promoter. [file 13100_2019_194_MOESM6_ESM.pdf]

### A. All full length L1s by subfamily

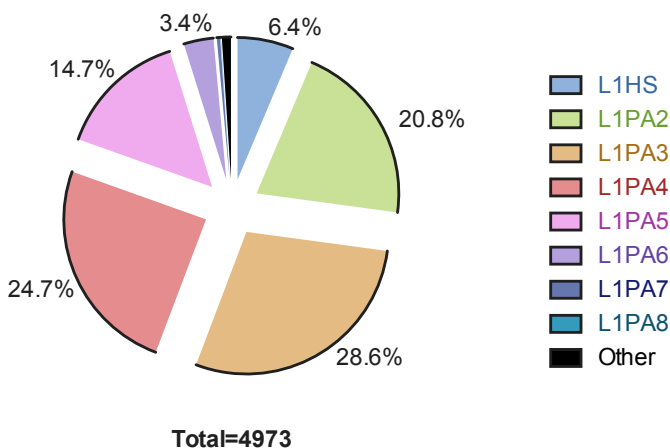

### B. Expressed L1s in whole cell

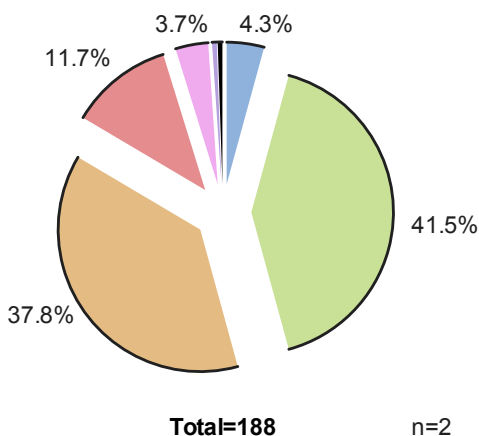

### C. Expressed L1s in cytoplasm

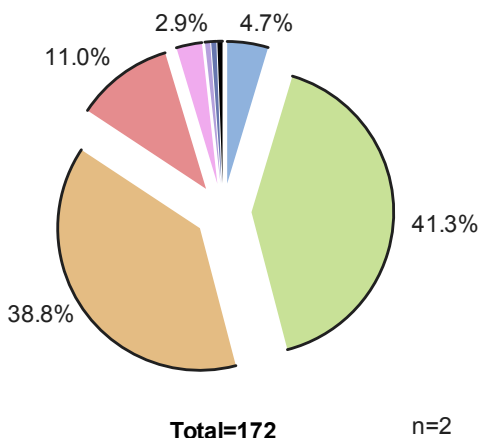

Supplement: Supplementary file 7 — Additional file 7: Figure S2. A) Subfamily distribution of full length L1 s in the human genome. B) Subfamily distribution of full length L1 s expressed in the whole cell preparation of 22Rv1 with n = 2. C) Subfamily distribution of full length L1 s expressed in the cytoplasmic preparation of 22Rv1 with n = 2. Colors are designated according to the legend by subfamilies L1HS, L1PA2, L1PA3, L1PA4, L1PA5, L1PA6, L1PA7, L1PA8, and Other. The other category includes L1MA4A, L1MA7, L1P1, L1P2, L1PA16, L1PA8A, L1 PB1, and L1BP4. Percentages of the L1 subfamilies are noted around the pie charts. [file 13100_2019_194_MOESM7_ESM.pdf]

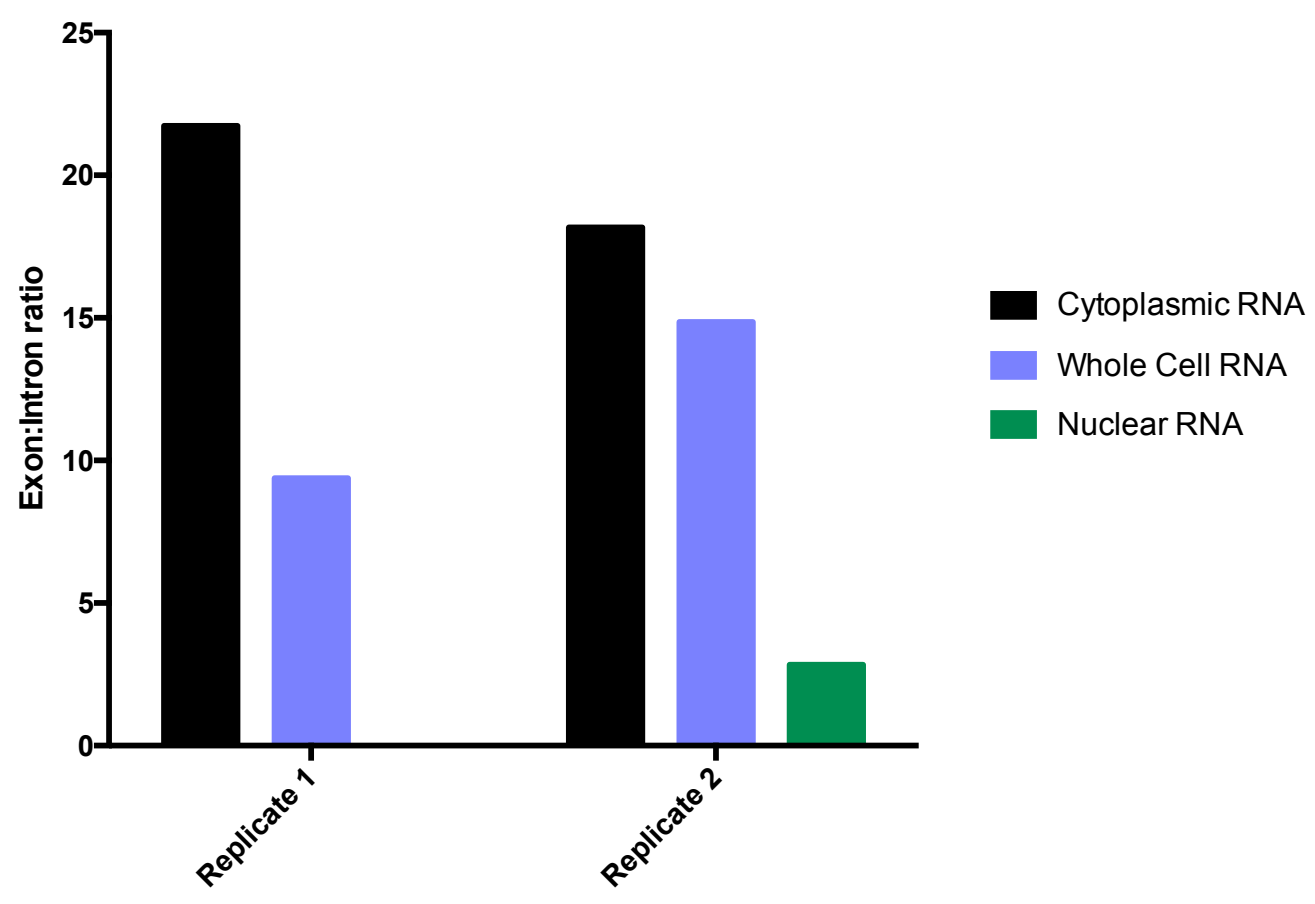

Supplement: Supplementary file 8 — Additional file 8: Figure S3. A) Estimated ratio of exonic reads to intronic reads in replicate 22Rv1 RNA-seq samples. The black bars represent the ratio of exonic to intronic reads in the cytoplasmic RNA samples, the purple bars represent the ratio of exonic to intronic reads in the whole cell RNA samples, and the green bar represents the ratio of exonic to intronic reads in the nuclear RNA samples. [file 13100_2019_194_MOESM8_ESM.pdf]

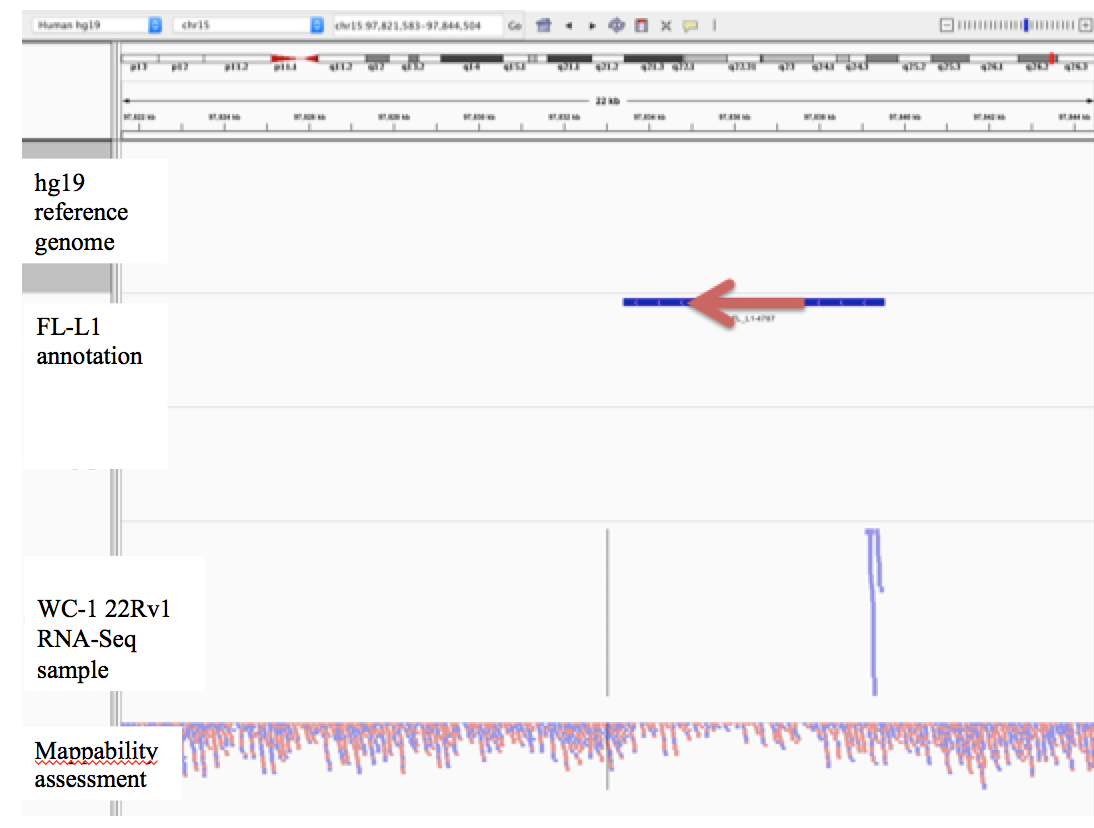

Supplement: Supplementary file 9 — Additional file 9: Figure S4. Example of L1 with antisense promoter activity de-coupled from sense promoter activity visualized in IGV. Loaded into IGV are the human reference genome, the human full-length L1 annotation, WC 22RvI bam file from replicate 1, and lastly the genomic HeLa bam file to assess mappability, which are all available upon author request. Arrows have been added to aid in the visualization of direction of the annotated L1. Arrows and reads in red are oriented in sequence from right to left. Arrows and reads in blue are oriented in sequence from left to right. [file 13100_2019_194_MOESM9_ESM.png]
